# Systematic review of the effective components of psychosocial interventions delivered by care home staff to people with dementia

**DOI:** 10.1136/bmjopen-2016-014177

**Published:** 2017-02-09

**Authors:** Penny Rapaport, Gill Livingston, Joanna Murray, Aasiya Mulla, Claudia Cooper

**Affiliations:** 1 Division of Psychiatry, University College London, London, UK; 2 Health Services and Population Research Department, Institute of Psychiatry, Psychology and Neuroscience, King's College London, London, UK; 3 UCLH CCTU (Haematology Trials), London, UK

**Keywords:** QUALITATIVE RESEARCH

## Abstract

**Objectives:**

This review aims to understand what elements of psychosocial interventions are associated with improved outcomes for people with dementia to inform implementation in care homes.

**Design:**

A systematic review of qualitative and quantitative intervention studies was undertaken.

**Eligibility criteria for included studies:**

We included primary research studies evaluating psychosocial interventions that trained care home staff to deliver a specific intervention or that sought to change how staff delivered care to residents with dementia and reported staff and resident qualitative or quantitative outcomes.

**Methods:**

We searched MEDLINE, PsychINFO and EMBASE electronic databases and hand-searched references up to May 2016. Quality of included papers was rated independently by 2 authors, using operationalised checklists derived from standard criteria. We discussed discrepancies and reached consensus. We conducted a narrative synthesis of quantitative and a thematic synthesis of qualitative findings to find what was effective immediately and in sustaining change.

**Results:**

We identified 49 papers fulfilling predetermined criteria. We found a lack of higher quality quantitative evidence that effects could be sustained after psychosocial interventions finished with no evidence that interventions continued to work after 6 months. Qualitative findings suggest that staff valued interventions focusing on getting to know, understand and connect with residents with dementia. Successful elements of interventions included interactive training, post-training support, aiming to train most staff, retaining written materials afterwards and building interventions into routine care.

**Conclusions:**

Psychosocial interventions can improve outcomes for staff and residents with dementia in care homes; however, many trial results are limited. Synthesis of qualitative findings highlight core components of interventions that staff value and feel improve care. These findings provide useful evidence to inform the development of sustainable, effective psychosocial interventions in care homes.

**Trial registration number:**

CRD42015017621.

**Strengths and limitations of this study**

- This is the first study to systematically review qualitative and quantitative studies which consider the impact of psychosocial interventions on care home staff and residents with dementia.
- By focusing on psychosocial interventions delivered either by training care staff to change their practices or interventions directly delivered by care home staff, our review informs development of sustainable interventions in ‘real-world’ care home settings.
- In reviewing such heterogeneous research studies, it was not possible to meta-analyse the quantitative findings.
- The qualitative papers report mainly on different interventions to those in the quantitative studies reviewed; therefore, we cannot conclude whether the intervention components staff reported as working well in qualitative studies were also associated with positive outcomes in the quantitative studies.

## Background

There are 850 000 people living with dementia in the UK and the numbers are increasing, as they are globally.^1^ Around 300 000 people in the UK live in care homes, about 70% of whom have dementia.^1^ Many have complex needs with high levels of neuropsychiatric symptoms^2^ associated with lower quality of life and higher care costs.^3^ ^4^ Public policy calls for high quality, evidence-based psychological interventions and an ‘informed and effective workforce’ to support people with dementia.^5^ ^6^ However, care home staff are often poorly trained and paid little with high staff turnover.^7–9^

Reviews considering the effectiveness of psychosocial interventions in care homes have drawn mixed conclusions, reflecting the diversity of interventions, objectives and outcomes.^10–14^ A recent systematic review of non-pharmacological management of agitation concluded that supervised interventions which promote better communication, interaction and understanding between care home staff and people with dementia, including dementia care mapping (DCM) and person-centred care (PCC), can reduce agitation immediately and for up to 6 months afterwards.^12^

Authors of a recent review of randomised controlled trials (RCTs) of non-pharmacological interventions for agitation and aggression in dementia, which included a narrower range of study designs, reported, in contrast, that overall, neither patient-level interventions (delivered directly to residents) nor care-delivery-level interventions (targeting how or the environment in which staff deliver care) were better than usual care in managing agitation and aggression.^10^ They concluded that existing evidence has troubling conceptual and methodological weaknesses, and that where individual studies show significant reductions in agitation, effect sizes are unlikely to be clinically meaningful.

Overall, although some psychosocial interventions are efficacious in managing specific neuropsychiatric symptoms in care home residents with dementia,^12–14^ positive effects are not sustained^10–12^ and rely on access to highly specialist external support.^13^ Additionally, there is little or no evidence of efficacy of stand-alone care home staff training unless ‘reinforcing’ (eg, additional supervision or individual skills training) or ‘enabling’ (time and help to put learning into practice) strategies are incorporated.^15–17^

To develop effective interventions for people with dementia living in care homes, we need to understand what works and how intervention effects can be sustained and embedded (ie, implementation) into practice after training. Quantitative reviews of efficacy in relation to defined outcomes can inform the former but have not until now informed the latter. Qualitative syntheses can inform implementation and translation of interventions from research into practice.^18^ Two existing studies have reviewed how psychosocial interventions for people with dementia in care homes have been implemented. The first (up to 2011)^19^ only reviewed qualitative studies, and the second (up to 2012)^20^ reviewed the effect of the interventions on staff knowledge, attitudes and skills but not resident outcomes.

Interventions are rarely implemented in the way they were carried out in trials, and findings of overall efficacy are generally conflicting.^10^ ^11^ ^13^ There is thus a need to understand which intervention components work, to inform real-world implementation. We have therefore (1) reviewed the evidence in quantitative intervention studies delineating what works immediately and where there is evidence of sustained effects on outcomes for people with dementia and care staff; and (2) synthesised qualitative research exploring what intervention components were considered to have worked by care home staff and other stakeholders and to have been practicable to implement. We intend that findings will inform the future development and implementation of sustainable psychosocial interventions.

## Methods

### Search strategy

We searched MEDLINE, PsychINFO and EMBASE with no restrictions on date or language of publication on 6 June 2014 and updated the search on 20 May 2016. We used the terms ‘care home’, ‘institution’, ‘24 hour care’, ‘residential home’, ‘nursing home’, ‘assisted living residence’ or ‘long-term care’ together with ‘staff’, ‘care worker*’, ‘nursing staff’, ‘care staff’, ‘care assistant*’ or ‘paid carer*’ and ‘intervention’, ‘training’, ‘staff training’, ‘staff education’ or ‘staff training intervention*’ combined with ‘dementia’, ‘Alzheimer’ or ‘vascular dementia’. References of included papers and relevant systematic reviews were hand searched for further papers (see online supplementary appendix 1 for a full search strategy).

10.1136/bmjopen-2016-014177.supp1 supplementary appendix

### Inclusion criteria

We included studies that fulfilled all the following criteria:

- Primary research.
- Quantitative with a control group (either individual or cluster RCTs or pre–post test studies with control conditions) or qualitative studies.
- Evaluating psychosocial interventions without significant medical or drug care element, for example, review by pharmacists or physicians.
- *Either* interventions that trained care home staff to deliver a specific intervention *or* that sought to change how care home staff delivered care to residents with dementia.
- Reporting staff and resident outcomes.

### Exclusion criteria

We excluded studies if:

- The intervention was delivered directly to older people by external health or social care professionals.
- Reporting on single-case studies and meeting abstracts.

PR read and screened titles and abstracts of studies. PR and CC independently read all retained papers. The decision to include or exclude papers was agreed by consensus.

### Assessment of quality

**Figure 1:**
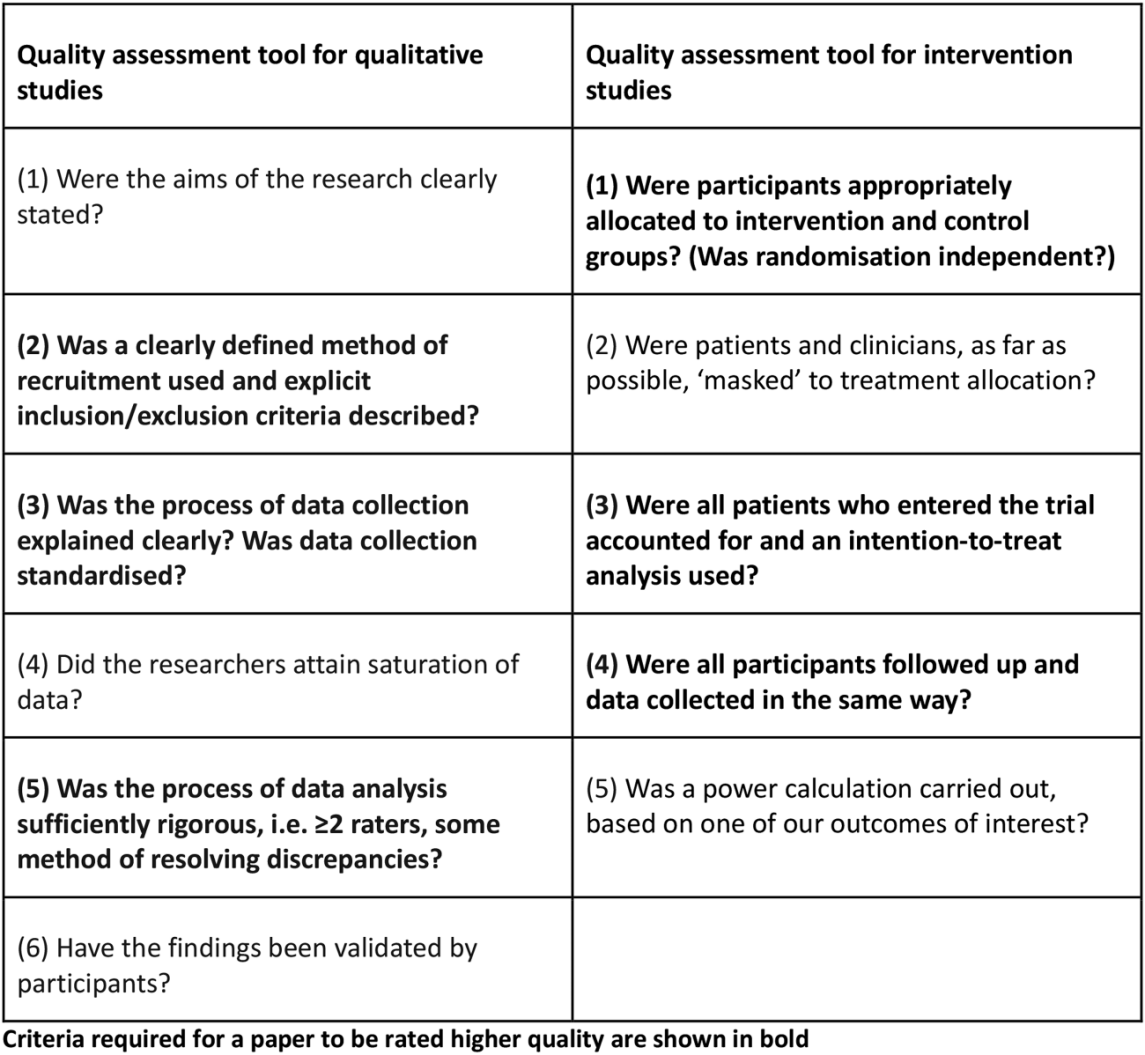
Tools used to rate validity of qualitative and quantitative studies. Adapted from Mukadam *et al*,^23^ Cooper *et al*^21^ and Lord *et al.*^22^

PR, CC and AM rated the quality of papers independently, using operationalised checklists and criteria for defining higher quality studies developed by our group^21–23^ from standard quality criteria^24^ (described in figure 1). Each quality checklist item scored 1 point; possible scores ranged from 0 to 6, with higher scores indicating better quality. We discussed discrepancies and reached consensus. For quantitative studies, we categorised papers as higher quality (ie, with a low risk of bias) if they: allocated participants to the intervention and control groups through independent randomisation, accounted for all participants who entered the trial and collected data and followed up participants in the same way (table 1, validity criteria 1, 3 and 4). For qualitative studies, we categorised papers as higher quality if they: used a clearly defined recruitment method, clearly stated inclusion and exclusion criteria, standardised data collection and involved two or more independent raters in data analysis (table 2, validity criteria 2, 3 and 5).

**Table 1:** Characteristics and quality ratings of high-quality quantitative studies

			n		Control	n		Validity criteria (see figure 1)				
Study	Recruitment source	Group training intervention	Staff	Resident		Staff	Resident	1	2	3	4	5
McCallion *et al*^39^	Residents with ≥1 problem behaviour and nursing assistants on two US nursing facilities	NA Communication Skills Programme; 5x 45 min didactic and interactive group (3–6 NAs) sessions, manual and videos; 4× 30 min individual, personalised training, practice and feedback. Individual make-up sessions offered. Monthly follow-up sessions with facilitator for 3 months. Delivered by Masters social worker	39	49	WLC crossover at 6 months (followed up at 9 months)	49	56	Y	Y	Y	Y	N
Pellfolk *et al*^51^	40 group-dwelling dementia units with high levels of restraint use	Restraint minimisation education. 1 person per unit attended 2 days training. 6× 30 min video lectures for all staff with units facilitating group discussion of 3 vignettes	156	149	WLC	133	139	Y	N	Y	Y	Y
Chenoweth *et al*;^55^ Jeon *et al*^56^	Staff and residents with needs-driven compromised behaviour in 15 Australian care homes using task-focused, not person-centred, care	PCC—2 days of training for 2 staff/site by experienced researchers+training manuals. Trained staff supported to develop and implement resident care plans. Regular telephone contact+2 visits during intervention. DCM—2 staff at each site, trained by expert, did DCM with researchers for 6 hours/day for 2 days; developed care plans and helped staff to implement them with regular phone support	56 45	77 95	TAU	23	64	Y	Y	Y	Y	Y
van de Ven *et al*^66^ van de Ven *et al*^63^	Nursing staff and residents with ≥1 NPS in 34 units in 11 Dutch nursing homes	Training staff to implement DCM. Managers selected 2 staff per home to train and each home had a DCM briefing day with specialists. The trained mappers then completed at least 2 DCM cycles	119	74	TAU	161	102	Y	N	Y	Y	Y

CG, control group; DCM, Dementia Care Mapping; IG, intervention group; NA, nursing assistant; PCC, person-centred care; TAU, treatment as usual; WLC, wait list control.

**Table 2:** Characteristics and quality ratings of high-quality qualitative studies

Study	Recruitment Source	Method	N	Type of intervention	Focus of analysis/key themes	Validity criteria (see **figure 1**)					
						1	2	3	4	5	6
Alnes *et al*^72^ Alnes *et al*^73^	Staff in 4 Norwegian dementia care units	Focus groups, semistructured interviews, analysis of recorded intervention sessions and log kept by trainer	24 staff participated in focus groups. 12 staff participated in semistructured interviews	MMC—a video-based counselling method to improve interaction skills. Staff received seven 1.5 hour weekly sessions over 2 months with an MMC trainer	Alnes *et al*—Nurses’ perception of learning from MMC. 2 overall themes were staff gaining new knowledge about themselves and the residents. Alnes *et al*—Factors that impact on learning outcomes of MMC intervention. Identified: (1) Establishing a common understanding of the content and form of MMC. (2) Ensuring that staff want to participate in and have the opportunity to do so. (3) Creating an arena for discussion and interactions during and after MMC	Y	Y	Y	N	Y	N
Figueiredo *et al*^75^ Marques *et al*^76^	Day staff in 1 Portuguese long-term care home	Pilot evaluation of staff training intervention included analysis of recorded morning care and postintervention focus group	6 staff took part in training and 5 participated in the focus group	8 psycho-educational sessions with staff with between session individual support. Intervention included staff support, multisensory stimulation and motor stimulation. Delivered by a multidisciplinary team and included homework and handouts	Figueiredo *et al*—Staff perspectives on structure and organisation and of benefits of the programme: (1) Acquisition of new knowledge and competencies. (2) Demystification of pre-existing beliefs. (3) Group cohesion. (4) Self-worth feelings. (5) Positive coping strategies. Marques *et al*—The impact of the motor and multisensory care-based approach on care practices, suggestions for future programmes, and difficulties putting into practice	Y	Y	Y	N	Y	N
Kontos *et al*^77^	Staff in 2 Canadian nursing homes	Postintervention focus groups and semistructured interviews.	14 staff participated in 2 focus groups and 10 staff were individually interviewed	12 week (2 hours each week) arts/drama informed educational intervention to improve person centred care. Used dialogue, critical reflection, role-play and dramatised vignettes	Staff perspectives on intervention. 2 main themes described: (1) Meaning beyond dementia—focused on how understanding behaviour facilitated care. (2) The influence of the approach to care—focused on how staff responses facilitate or inhibit person-centred care	Y	Y	Y	N	Y	N
Veraik *et al*^67^	Staff in 9 wards in Dutch nursing homes from an RCT intervention group	Semistructured interviews, questionnaire data and analysis of minutes, session reports and observations	98 CNAs were trained. 20 CNAs were interviewed including 10 most and 10 least positive about the intervention	Guidelines for managing depression in dementia. Included: Printed educational materials, three interactive team training sessions and setting up promotion group on each ward	Analysed data from successful, moderately successful and unsuccessful implementation sites and analysed at multiple levels, nursing home, ward, CNA and resident levels. Presented case studies of successful/unsuccessful implementation and factors influencing successful introduction and application of the guideline intervention	Y	Y	Y	N	Y	N

CNS, certified nursing aides; MMC, Marte Meo counselling.

### Synthesis and analysis of data

In our narrative synthesis of quantitative studies, we prioritised results from higher quality studies and findings on primary outcome measures. Results from lower quality quantitative studies are included in online supplementary table S1. As in our previous work,^12^ we decided a priori to meta-analyse when there were three or more RCTs investigating sufficiently homogeneous interventions and outcomes. No intervention met these criteria. There are no commonly agreed criteria for excluding qualitative studies based on quality;^25–28^ therefore, we included all qualitative studies in our ‘thematic synthesis’ of qualitative findings, in line with previous similar reviews^19^ ^28^ and accepted methods.^26^ ^29^ PR extracted data from the qualitative papers' results sections into NVIVO 9 software and inductively coded it in an open-ended, exploratory manner. CC reviewed the data and the coding frame; differences were discussed and codes refined. We then related our descriptive themes to our question of what components of interventions were considered to have worked and to have been practical to implement.^30^ ^31^ PR developed overarching themes that synthesised the evidence and CC further refined emergent themes.

## Results

**Figure 2:**
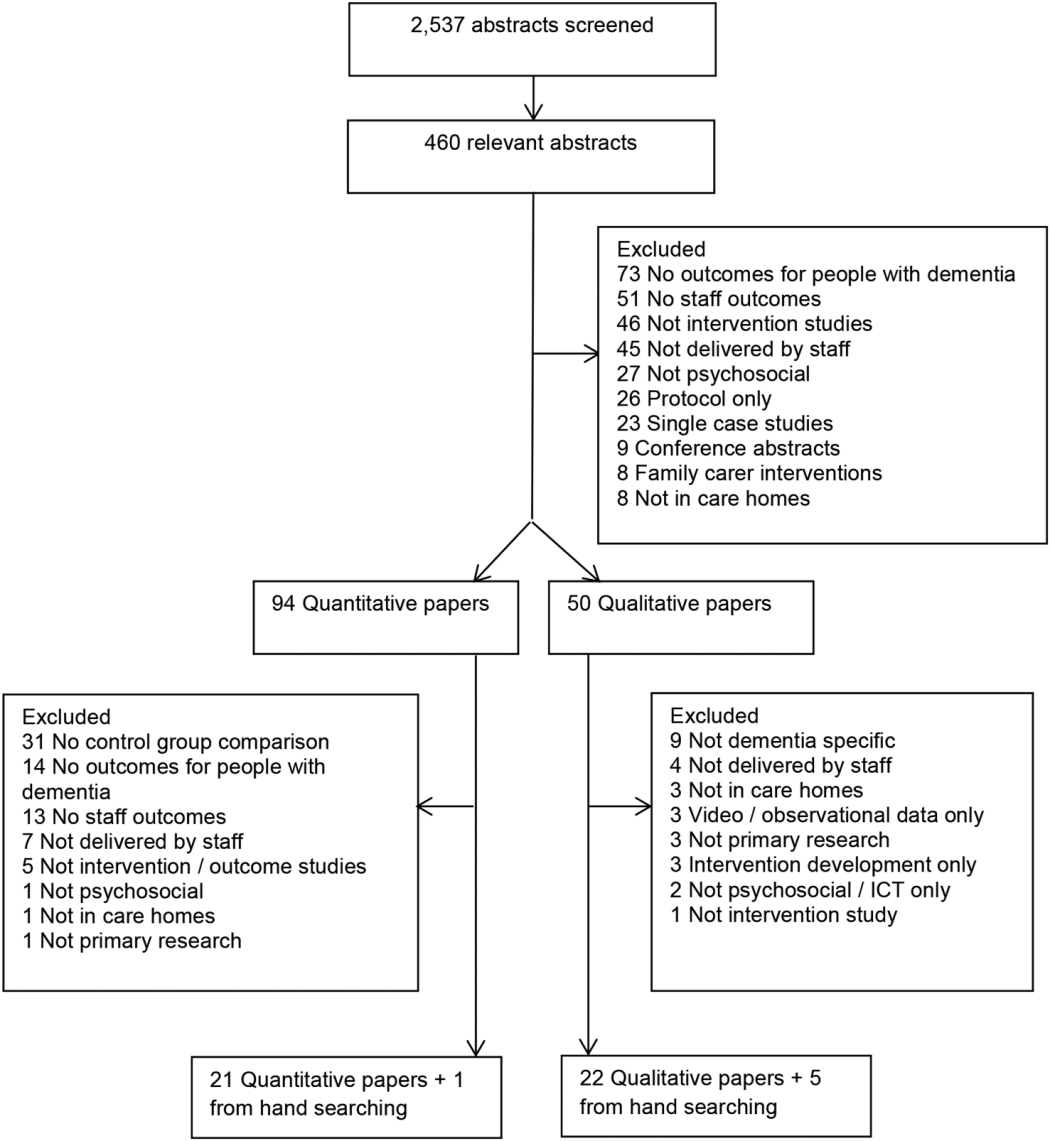
PRISMA diagram.

We identified 2537 unique, potentially eligible studies and included 49 relevant papers (see Prisma diagram figure 2 and online supplementary appendix 2 for the PRISMA checklist). We categorised 6 of the 27 qualitative papers and 6 of the 22 quantitative papers as higher quality. The relevant studies took place in the USA,^32–43^ Sweden,^44–52^ Australia,^53–60^ the Netherlands,^61–67^ the UK,^68–70^ Norway,^71–74^ Portugal,^75^ ^76^ Canada,^77^ ^78^ Ireland^79^ and Germany.^80^ They describe diverse interventions, including training and delivery of person-centred and relationship-focused care and DCM,^36^ ^37^ ^40^ ^43^ ^44^ ^52^ ^53^ ^55–57^ ^59^ ^63^ ^66^ ^68^ ^72–74^ ^78^ ^79^ training in dementia and managing difficult behaviour,^41^ ^42^ ^58^ ^60^ ^80^ communication skills and awareness training,^32–34^ ^38^ ^39^ ^62^ ^64^ ^69^ creative and sensory interventions,^45–47^ ^61^ ^65^ ^70^ ^71^ ^75–77^ staff supervision interventions,^35^ ^48–50^ restraint minimisation^51^ and behavioural therapy interventions.^67^

10.1136/bmjopen-2016-014177.supp2 supplementary appendix

### Findings from high-quality quantitative studies

The higher quality quantitative papers are described in table 1, and the lower quality quantitative papers are described in online supplementary table S1.

10.1136/bmjopen-2016-014177.supp3 supplementary tables

#### Group training interventions for care home staff with additional individual supervision

We identified one high-quality study that included individual skills training in addition to group training for nursing assistants.^39^ The training was designed to increase knowledge of dementia, communication and management of problem behaviours. It was tested in two US nursing homes in a crossover RCT. Resident physically aggressive behaviour in the intervention group decreased 3 months postintervention (*F*=17.59, p<0.001) relative to the control group, but this was not maintained at 6 months. However, verbally aggressive (*F*=14.23, p<0.001) and depressive symptoms (p<0.05) were significantly lower in the intervention group than the control group 6 months postintervention.

#### DCM interventions

Four papers described two high-quality RCTs^55^ ^56^ ^63^ ^66^ evaluating DCM, a multicomponent, person-centred intervention. CADRES (Caring for Aged Dementia Care Resident Study)^55^ ^56^ compared PCC and DCM with usual care in a three-arm RCT in 15 Australian care homes providing task-focused care. The DCM intervention included systematic observations of the well-being of people with dementia categorised and fed back to staff to support PCC. The mapping was completed by study experts and by trained care home staff. At the 4 month follow-up, resident agitation was lower in the DCM (10.1, 95% CI 0.7 to 21.1; p=0.04) and PCC (13.6, 95% CI 3.3 to 23.9; p=0.01) groups compared with the intervention group. Among staff, at the 4 month follow-up on three subscales of the Maslach Burnout Inventory (MBI), emotional exhaustion was lower in the DCM group than in the PCC and control groups (*F*=2.77, p=0.03), but there was no significant difference in depersonalisation or personal accomplishment. In another high-quality study which tested DCM in less tightly controlled settings, with care home staff delivering more of the intervention and without recruiting task-focused homes, no significant differences were identified between the intervention and control groups on primary staff or resident outcomes.^63^ ^66^

#### Group training interventions for care home staff without additional supervision

A cluster RCT evaluated^51^ a restraint minimisation group training programme without additional supervision in 40 Swedish dementia units. Immediately postintervention, residents in the intervention group were restrained less than those in the control group (OR=0.35, 95% CI 0.15 to 0.83, p=0.02). Among staff who received the intervention, knowledge of restraint use (p=0.02) and dementia (p=0.01) increased significantly compared with staff in the control group, but there was no difference in staff attitudes towards restraint use. Longer term outcomes were not reported.

### Findings from qualitative studies

We have synthesised findings from all included qualitative papers with at least one higher quality paper contributing to each main theme, with higher quality studies contributing to more subthemes than lower quality studies. The findings from the high-quality studies are presented in table 2 and findings from lower quality qualitative papers are presented in online supplementary table S2.

#### What works? Beneficial components of interventions

##### Improving communication

Staff across diverse studies described practices that improved interaction and communication with residents with dementia.^37^ ^44–49^ ^52^ ^53^ ^59^ ^65^ ^68^ ^70–72^ ^75^ ^76^ ^78^ ^79^ These included interventions that focused on: initiating ‘meaningful conversation’ with residents during care;^52^ ^53^ ^68^ ^75^ the emotional content of interactions,^44–48^ ^52^ ^68^ ^71^ touch and physical contact,^49^ ^52^ ^53^ ^71^ ^72^ ^78^ maintaining eye contact and using simple clear instruction.^44^ ^47^ ^72^ ^76^ ^78^ In addition to improvements in their own communication, staff described positive changes in residents' responses, noticing they were more responsive, happier and more cooperative.^45^ ^47^ ^52^ ^53^ ^79^ Giving residents time and space to respond was perceived as beneficial.^48^ ^49^ ^52^ ^53^ ^65^ ^71^ ^72^ ^78^ Staff observed that by taking time to understand residents' responses, residents seemed more able to make decisions and actively participate in their care. Staff who participated in singing interventions^45–47^ found themselves talking and instructing less with residents understanding and expressing themselves more effectively.

##### Enhanced understanding of the residents

Staff reported that interventions enhanced their understanding of the residents.^37^ ^44–50^ ^52^ ^53^ ^57^ ^59^ ^61^ ^65^ ^67^ ^68^ ^70–73^ ^75–79^ They felt more able to put themselves ‘in the client's shoes’^78^ and empathise with people with dementia,^48–50^ ^52^ ^53^ ^57^ ^59^ ^68^ ^72–75^ ^77–79^ which was intrinsically rewarding.^48^ ^59^ Staff reflected that this extended to understanding relatives' perspectives,^48^ ^53^ ^59^ resulting in improved relationships between staff and relatives.^53^ ^59^ ^79^

Staff across a range of studies recognised the importance of getting to know the person with dementia in order to provide more individualised and ‘person-centred’ care.^45^ ^48^ ^49^ ^52^ ^53^ ^59^ ^68^ ^70^ ^71^ ^77–79^ This was achieved both by engaging people with dementia in activities where they could express their individuality such as dancing, singing and sensory activity;^45–47^ ^70^ ^71^ and through interventions which encouraged staff through training, supervision and experiential learning to find out more about care recipients.^48^ ^50^ ^53^ ^59^ ^68^ ^77^ Developing staff knowledge of residents facilitated their understanding of the potential meaning of residents' behaviours, enabling them to alter their responses accordingly.^37^ ^44^ ^52^ ^53^ ^59^ ^68^ ^71–73^ ^77^ ^79^ Staff identified this as important for identifying residents' strengths and weaknesses^48^ ^49^ ^53^ ^57^ ^59^ ^72^ ^75–77^ ^79^ and promoting independence when providing care.^45–48^ ^52^ ^53^ ^61^ ^70^ ^71^ ^77–79^

##### Reflection facilitates good practice

A common process underlying improved communication and understanding is an emphasis within interventions on staff reflecting on their practices. Staff appreciated the opportunity to consider their own and residents' interactions within experiential learning,^77^ interactive training,^59^ ^75^ ^76^ formal supervision^48–50^ ^71^ or video feedback.^37^ ^41^ ^44^ ^46^ ^48–50^ ^52^ ^71^ ^72^ ^75–78^ This enabled them to identify patterns in their own and residents' behaviours,^44^ ^48^ ^49^ ^59^ ^71^ ^72^ consider alternative reactions,^44^ ^48^ ^49^ ^71^ ^72^ ^77^ and feel validated about helpful practices while recognising unhelpful practices and assumptions.^41^ ^59^ ^67^ ^76^ ^78^ ^79^

#### Barriers and facilitators: individual factors

##### What gets in the way?

Staff across studies described the negative impact of providing care, particularly personal care, to people with dementia on themselves and their feelings about work.^46^ ^49^ ^50^ ^52^ ^71^ When faced with resistance and verbal and physical aggression, staff described frustration and distress.^41^ ^46^ ^47^ ^49^ One carer described this struggle: “I wonder how long you can do this. … It is hard to ﬁght every morning and only get anger back. … What should we do, we just have to live with it, right? I hide in the laundry room to catch my breath before caring for her.”^46^

Staff were sometimes reluctant to engage with interventions. For some, interventions promoting emotional and physical closeness led to fears of becoming attached to residents.^44^ ^52^ ^70^ Staff expressed doubts about their own ability to implement interventions,^37^ ^44^ ^45^ ^49^ ^53^ ^61^ ^70^ ^73^ ^74^ either in terms of having specific skills, such as being able to sing,^45^ ^61^ or having the confidence to take on new roles, such as approaching relatives^37^ ^53^ or coordinating care.^74^ There was initial scepticism from staff about engaging with interventions, especially if they were perceived to involve additional work, changes to existing ways of working^45^ ^49^ ^53^ ^59^ ^65^ ^67^ or unfamiliar techniques.^44^ ^49^ ^61^ ^73^ Negative responses towards interventions were more apparent when staff felt they did not accommodate the varying levels of education and experience within a team^49^ ^53^ ^67^ ^72^ ^73^ or the complex needs of those they cared for.^52^ ^61^ ^67^ ^74^

##### What makes it easier?

A key facilitator of staff engagement was seeing benefits for staff and residents rather than being told of potential benefits by trainers, especially when staff saw positive changes in residents.^37^ ^45^ ^46^ ^48^ ^52^ ^53^ ^57^ ^59^ ^61^ ^65^ ^67^ ^70^ ^72^ ^75^ ^76^ ^79^ In numerous studies, staff observed decreased agitation and aggressive behaviours, which they associated with the interventions.^45^ ^47^ ^52^ ^53^ ^65^ ^70^ ^79^ Staff identified a link between the impact of interventions on residents, and fewer difficulties providing care, a calmer and more relaxed atmosphere and improved relationships with residents and relatives.^37^ ^44–46^ ^48–50^ ^52^ ^53^ ^57^ ^59^ ^61^ ^65^ ^68^ ^70^ ^71^ ^75–79^

Having the opportunity to reflect on and adapt practices, using active and interactive learning methods was central to a number of interventions. Staff reported that group-based activities facilitated discussion and shared learning within teams^57^ ^67^ ^75^ and that role-play, the use of vignettes and analysis of filmed interactions supported understanding.^44^ ^49^ ^61^ ^75^ ^77^ Access to written materials including manuals, tip-sheets and hand-outs was valued when clearly written to accommodate the educational level of the staff.^52^ ^57^ ^74^ ^75^

#### Barriers and facilitators: social and team factors

##### What gets in the way?

Lack of cooperation within teams was cited as a barrier to implementation, with staff identifying colleagues' unwillingness to help each other and poor communication as obstacles.^65^ ^67^ ^76^ ^78^ Staff reported difficulties sharing new approaches with staff who had not attended training, especially those who had opted not to participate or held negative attitudes.^44^ ^53^ ^59^ ^67^ ^73^ ^74^ Staff did not wish to be seen as telling colleagues what to do or felt that they lacked authority to do so.^59^ ^67^ ^73^ ^74^ Lack of ownership of new interventions within the care team was cited as a barrier to initial implementation^44^ ^53^ ^61^ ^65^ ^67^ ^74^ ^78^ and maintaining positive changes after research trials.^53^ ^59^ ^61^ This was noted when staff felt that changes were imposed in a top-down way by managers or external professionals.^53^ ^67^

##### What makes it easier?

Participants suggested that all staff should be included in training or new interventions to promote learning and help sustain practices.^44^ ^52^ ^53^ ^59^ ^67^ ^73^ ^76^ ^78^ Staff also valued the opportunity to share learning within teams.^44^ ^53^ ^57^ ^61^ ^65^ ^67^ ^73–75^ ^77^ Some interventions included formal structures, such as a ‘digital database’ for sharing ideas,^61^ or structured ‘consensus meetings’ led by team members, while others built discussion into existing forums or had informal discussions during routine care.^65^ ^67^ ^74^

Common across studies was the importance of on-site support to put skills into practice.^53^ ^57^ ^61^ ^65^ ^67^ ^73^ ^75^ This reinforced learning and gave staff opportunities to refine strategies and troubleshoot. Most studies included some support outside of formal training either as supervision and direct feedback on care^37^ ^41^ ^44^ ^48–50^ ^52^ ^61^ ^71–76^ or through on-site mentoring.^37^ ^53^ ^57^ ^59^ ^65^ ^67^ ^78^ Having on-site mentors trained as part of the intervention has the benefit of being sustainable postintervention but relies on committed individuals within the home who require additional support.^37^ ^53^ ^59^ ^65^ ^67^

#### Barriers and facilitators: organisational factors

##### What gets in the way?

Lack of time was raised as a barrier across most studies in relation to finding time to attend training and supervision and put learning into practice.^44^ ^52^ ^65^ ^73^ ^74^ ^79^ When interventions required staff to set up additional project meetings, it was noted that these happened infrequently^65^ ^74^ and more intensive interventions, requiring additional activities, such as detailed care plans and indepth observation, were difficult to sustain,^44^ ^52^ ^53^ ^61^ ^65^ ^67^ ^74^ particularly when staff felt that research teams were unclear about the time commitment required.^44^ ^61^ Staff identified incompatibility between their busy, pressurised shifts and interventions that required them to engage with residents at a slower pace, shifting from a task-focused to a relationship-centred approach.^44^ ^59^ ^78^ ^79^ High staff turnover and low staffing ratios were also barriers. In addition to an increased workload, lack of consistency in staffing resulted in less opportunity for shared learning, less coordination within teams and less familiarity with residents.^48^ ^53^ ^61^ ^65^ ^67^ ^73^ ^76^ ^78^

Parallel change, such as organisational restructuring, new IT systems or new training initiatives were seen to hinder implementation.^59^ ^67^ ^78^ Although management and care home policy promoted a ‘person-centred’ approach, in practice staff felt that task completion remained a priority for managers and peers.^41^ ^44^ ^53^ ^59^ ^67^ ^70^ One staff member commented: “I would rather be doing my care plans…because that is probably judged by others, whereas the project is not judged.”^59^ When staff felt unsupported by management, they found it difficult to prioritise new ways of working^53^ ^57^ ^59^ ^61^ ^65^ ^70^ ^76^ and teams were unmotivated when they felt they lacked the power to implement changes.^67^ ^73^ ^74^

##### What makes it easier?

Staff noted that management engagement with new interventions through attending training, contributing to project meetings or arranging cover for staff participation had positive effects,^53^ ^65^ ^67^ ^73^ ^74^ but in most studies, this was not the case. Being able to build the interventions into routine care was reported as central.^44–46^ ^48^ ^61^ ^65^ ^68^ ^70^ ^75^ ^78^ ^79^ Spending time talking to residents about their interests, reminiscing, singing to them or putting on a resident’s jewellery did not require additional time or resources and often made care provision more enjoyable for all.^41^ ^45^ ^48^ ^68^ Sharing information via booklets left in a resident's room or in team discussions resulted in new strategies being sustained without requiring major changes to existing practices.^48^ ^65^ ^68^ Interventions consistent with existing approaches were valued.^41^ ^49^ ^59^ ^61^ ^65^ ^67^ ^75^ ^78^ ^79^ Benefits were reinforced when staff felt that giving more time to engage residents, rather than rushing to complete tasks, saved time overall as residents were more engaged, cooperative and less distressed.^41^ ^49^ ^52^ ^53^ ^61^ ^65^ ^72^ ^77^ ^78^

## Discussion and conclusions

### Key findings

We found a paucity of higher quality evidence that effects could be sustained after care home psychosocial interventions finished and there was no evidence that any interventions continued to work after 6 months. In one higher quality study, an individual and group programme with monthly follow-up sessions^39^ decreased resident physical aggression after 3 months and resident depressive symptoms and verbal aggression up to 6 months later. This may relate to their inclusion of monthly top-up sessions in addition to group and individual skills training, highlighting the benefits of ‘reinforcing’ strategies.^15^ This is consistent with our qualitative findings. Staff found individualised support to put new approaches into practice and to sustain beneficial interventions. In one higher quality trial,^51^ training staff champions to implement a video case vignette training programme increased staff knowledge and decreased restraint use immediately; while evidence for DCM and PCC was mixed, with positive findings from an Australian study^55^ ^56^ not replicated in a more pragmatic, real-world care home environment.^63^ ^66^

The findings from the lower quality studies were consistent with our conclusions from higher quality studies. They were, however, more heterogeneous in terms of outcomes, type and intensity of interventions and study designs. Lower quality interventions offering no follow-up supervision or support demonstrated no effect on resident symptoms. Interventions which included individual skills training or supervision in addition to didactic group-based training were associated with reduced resident neuropsychiatric symptoms and improved care delivery skills among staff. In our qualitative synthesis, consistent with previous reviews,^14^ ^19^ we found that staff valued interventions that encouraged staff to get to know, understand and connect with residents with dementia. Interventions perceived as too intensive and complex for staff to put into practice, or as separate from rather than building on existing practices, were difficult to sustain. Staff described a number of beneficial ‘enabling’ practices such as having on-site mentors and opportunities to share new learning.

### Implications for clinical practice

Sustaining effects of psychosocial interventions in real-world care home environments after research teams move on is challenging and rarely accomplished. Our qualitative synthesis highlighted the components and characteristics of interventions that staff considered important for achieving this. Interventions should be interactive and staff should retain materials after the groups are finished. Focusing on the benefits of the interventions for staff, residents and their relatives within training and giving staff opportunities to experience the impact of interventions by practising skills between sessions and reflecting on what works may motivate staff to continue to use and embed skills in routine care. Interventions need to fit into day-to-day care, avoid lengthy record-keeping or intensive observations and should save more time than they take. Including management in training and holding separate sessions with management and senior staff can support implementation. Having management support to train all staff is likely to make the role of on-site mentors more achievable, increasing shared responsibility across teams.

### Strengths and limitations of this review

We reviewed studies testing a broad range of interventions, using qualitative and quantitative methods. This heterogeneity meant that it was not possible to meta-analyse quantitative data. By only including quantitative studies that report outcomes for staff and residents, we have excluded high-quality RCTs that may have provided further insights into the questions being addressed. However, without considering the effects of interventions on residents and staff, it is difficult to understand how altering staff practices impacts on care home residents.

The included qualitative papers report on interventions that were largely different from those in the quantitative studies reviewed, although there was overlap in the nature of the interventions. We cannot therefore conclude whether the intervention components staff reported in qualitative studies to work well were also associated with positive outcomes in the quantitative studies. However, staff training and support interventions would only be expected to ‘work’ if staff or home management change practice, and managers and staff generally only adopt new ways of working if they believe they make life better for the home, the staff or the residents. Consequently, qualitative studies that ask care home staff what components of interventions improved care delivery and how, provide useful evidence in an area where many trial results have been disappointing.

### Future research

Within this review, we have highlighted some of the beneficial intervention components and the potential barriers and facilitators to implementing psychosocial interventions in care homes. To fully understand what works in dementia care, studies need to report fully on the process of implementation, including full reporting on adherence and treatment fidelity, using a combination of qualitative and quantitative measures.^81^ ^82^ Very few of the quantitative studies gave details on attendance at sessions, how accurately staff were picking up new skills or how much staff were applying new learning or included any qualitative exploration of the process. Future RCTs in this area should consider implementation strategy from the outset and can draw on these findings to address the inherent challenges of embedding psychosocial interventions into care home settings.^82^
