# Supplementary material for: Systematic review of the effective components of psychosocial interventions delivered by care home staff to people with dementia
Source: BMJ Open. 2017 Feb 9;7(2):e014177. doi: 10.1136/bmjopen-2016-014177 (PMC5306506; doi:10.1136/bmjopen-2016-014177)
Supplement: supplementary appendix [file bmjopen-2016-014177supp_appendix1.pdf]

Supplementary appendix 1: Search strategy for Medline, PsychINFO and EMBASE (OVID) databases

We searched Medline, PsychINFO and EMBASE (OVID) databases for the following search terms (in any field) with no restrictions on date of publication or language of publication:

{care home} OR {institution} OR {24 hour care} OR {residential home} OR {nursing home} OR {assisted living residence} OR {long-term care}

AND

{staff} OR {care worker*} OR {nursing staff} OR {care staff} or {care assistant*} OR {paid carer*}

AND

{Intervention} OR {training} OR {Staff training} OR {staff education} OR {staff training intervention*}

AND

{Dementia} OR {Alzheimer} OR {Vascular dementia}
