# Supplementary material for: Systematic review of the effective components of psychosocial interventions delivered by care home staff to people with dementia
Source: BMJ Open. 2017 Feb 9;7(2):e014177. doi: 10.1136/bmjopen-2016-014177 (PMC5306506; doi:10.1136/bmjopen-2016-014177)
Supplement: supplementary tables [file bmjopen-2016-014177supp_tables.pdf]

Table 1: Methodological characteristics and quality ratings of lower quality quantitative studies

Study	Recruitment source	Group training intervention	n		Control	n		Significant differences in outcomes (Intervention group compared to control)				Validity (answers to questions 1-5)**				
			staff	resident		staff	resident	Staff – Immediate outcomes	Staff – longer term outcomes	Resident – immediate outcomes	Resident – longer term outcomes	1	2	3	4	5
**Borgeois et al 2001; Borgeois et al 2004; Burgio et al 2001**	Seven Florida nursing homes	Multi component intervention:<br>1. 2 hour didactic training on effective communication and memory aids by Clinical Psychologist.<br>2. One-on-one skills training research assistants.<br>3. Use of memory books with residents<br>4. A staff self-monitoring and supervisory feedback system.<br>5. Maintenance visits by project manager following intervention.	57 NAs and 23 qualified nurses	63	TAU	69	62	NAs communication skills improved PI (F(2,124) =17.20, p = .0001)<br><br>NAs used more positive statements PI F(2,124)= 6.16, p= .004<br><br>NAs talked more PI (F (1, 64) = 5.22, p < .05)	NAs improved communication skills (F(1,27) = 12.92, p= .001) and increased use of positive statements (F(1,27) =11.91, P=.002) were maintained at 4 month follow up	The rate of positive interactions between residents and staff increased PI (F(2,130) = 4.81, p = .01).<br><br>Residents with memory books talked more F (1, 64) = 8.96, p < .01) and perseverated less PI (F (1, 64) = 5.14, p< .05).	The increased rate of positive interactions between residents and staff was maintained at 4 month follow up (F(1,41) = 5.83, p=.02)	Y	N	N	Y	N
**Burgio et al, 2002**	Residents with disturbed behaviour and staff in nine units in two US nursing homes offering 5 hours of Behavioural and communicat	Formal staff management behavioural supervision by trained unit supervisors.	46 Certified Nursing Assistants	47	No additional supervision	39	32		At 3 month follow up staff were less likely to prompt multiple activities (F(1, 64)= 4.74, p= .05)<br><br>At 6 month follow up staff announced more single activities (F(1, 56)=6.22, P=.05), and more often delayed physical assistance following instruction			Y	N	N	Y	N

	ion training by a psychologist								(F(1, 56) = 6.49, p= .05).<br><br>At 6 months NAs used more positive statements during care interactions (F(1, 33)=5.33, p= .05.) but overall NAs interacted less frequently with residents (F(1, 45)=5.72, p=.05)							
**Clare et al, 2013**	Six specialist dementia and two mixed Welsh care homes	Two 90 minute training sessions in understanding awareness in severe dementia and using an observational measure + fortnightly group + access to weekly individual supervision over 8 weeks	32	32	TAU	33	33		No long term follow up	**Residents had better quality of life as rated by family members on QUALID (F1,29 = 5.88 p=0.02).**	No long term follow up	Y	Y	N	Y	Y
**Davison et al, 2007; Visser et al 2008**	Staff and residents with challenging behaviour in two high and two low level care facilities in Australia (three in Visser et al 2009)	Dementia Training program: 8 x 60-90 minute didactic and experiential sessions by experienced mental health clinicians<br><br>Training (as above) + 5x 30-60 minute informal group peer support sessions facilitated by research team.	35<br><br>29	46<br><br>35	WLC	26	32	Self-efficacy increased in both IGs vs CG PI (F(1,86)=23.74, p<0.001)<br><br>Perceived skills and knowledge improved in education + peer support group PI (F(2, 47)=6.10; p<0.001)	Increase in self-efficacy maintained at 6 month follow up (F(1,61)=5.07, p<0.05) with no additional effect of peer support PI or at follow up<br><br>Improved perceived skills and knowledge in education + peer support group was maintained at 3 (F(1, 16)=49.3; P<0.001) and 6 (F(1, 13)=21.7; p<0.001) month follow up.			Y	N	N	Y	N
**Finnema et al, 2005**	Nursing assistants and residents (not in need	9 months of emotion-oriented care: All staff trained (2 days, 2 weeks apart +	46	67	Dutch 'model care plan' alone.	53	79	Only found significant effects in subgroup analyses		Only found significant effects in subgroup analyses		Y	Y	N	Y	N

	of nursing) in 14 Dutch homes not using emotion-oriented care. Staff trained in Dutch 'Model care plan': training + adviser on unit, supervision + network meetings.	homework in between); advanced course for five staff/ward, advisor course for 1 staff/ward; 4 days of unit supervision on implementation.													
**Kuske et al, 2009**	Six German nursing homes	13 x 1 hour didactic and active learning weekly sessions on dementia care by a nursing and health scientist with practical experience.	38	68	WLC<br><br>13 x 1 hour weekly relaxation sessions by clinical psychologist.	28<br><br>30	74<br><br>68	**Knowledge of dementia increased (F=10.4, p=0.002) Perceived competence in dementia increased (F=3.7, p=0.056).**	**Increase in perceived competence maintained at 6 months (F=7.93, p=0.006) but knowledge was not.**		Use of restraint increased more in WLC (p=0.045)* and relaxation CG (p=0.038)* compared with IG over 6 months from similar baseline levels	Y	N	N	Y	N
**Magai et al, 2002**	Three US nursing homes.	10 x1 hour psychologist training / experiential sessions over 2 weeks training staff to recognise non-verbal and emotional signals. Individual make-up sessions offered.	9	41	WLC<br><br>Attentional control – 10x1 hour training sessions in behavioural and cognitive aspects of dementia.	7<br><br>5	27<br><br>23				Significantly more positive facial expressions observed 6 weeks post intervention (F=2.3, p<0.05) but not sustained at 9 or 12 weeks.	Y	Y	N	N	N

**Moyle et al, 2016**	Resident/ relative dyads and staff in four Australian LTC facilities	12 hours of didactic group training for staff and family members in 'capabilities model of dementia care' + two staff from each intervention site trained as 'capability mentors', support and mentoring offered to staff. Training delivered by registered nurses	51	37 (relative/ resident dyads)	4 hour training in PCC	30	11 (relative/ resident dyads)		12 months post intervention staff in the CG had significantly lower levels of job satisfaction than staff in the IG ($F(1, 68) = 8.42$, $p = 0.005$).		12 months post intervention residents in the CG had lower quality of life as rated by family members on QOL-AD than residents in the IG ($F(2, 92) = 3.99$, $p = 0.02$)	N	Y	Y	Y	N
**Sloane et al, 2004; Hoeffer et al, 2006**	Nursing assistants and residents agitated during bathing in fifteen US nursing homes.	A. Person centred showering<br>B. Person centred in bed towel bath with no rinse soap. Intervention groups crossed over at six weeks. Clinical nurse specialist or psychologist trained nursing assistants 2 days / week for 4 weeks with videotaping and live supervision.	24 (across both groups)	Group A 24<br><br>Group B 22	WLC	13	23	Increased use of gentleness and verbal support and in perceptions of ease in both IGs ($p=0.05$)*. Confidence increased in towel bath then showering group ($p=0.05$)*.	No long term follow up	**PI agitation and aggression ($p=0.02$)* and resident discomfort ($p=0.001$)*** decreased and skin condition improved in both IGs ($p<0.003$)*. Decline in discomfort significantly greater in towel bath than showering intervention.	No long term follow up	Y	Y	N	Y	N
**Sprangers et al, 2015**	Residents with dementia and nursing aides in one Dutch Nursing home	Communication skills training + individual observation and feedback on morning care. Two training sessions provided to staff with lower baseline communication skills and one training session given to staff with higher baseline communication skills.	24 across control and intervention group	26 across control and intervention group	TAU	N/A	N/A	Caregiver distress decreased post intervention ($F(1,24)=5.20$, $P<0.05$)	No longer term follow up		No longer term follow up	Y	N	N	Y	N

**Teri et al, 2005 (feasibility and RCT)**	Day staff and residents with problem behaviours in four US assisted living facilities.	STAR, a manualised dementia-specific 2 month staff training for with two half-day group workshops and four individual sessions. Didactic and interactive. 3 meetings for managers. Delivered by clinical psychologists and then graduate students.	25 across intervention and control group	31 across intervention and control group	TAU	N/A	N/A	IG reported less impact from resident problems NPI - staff impact (Z=-2.28 p<.022), RMBPC – Staff reaction (Z=-3.47, p<0.001).	No long term follow up	Behavioural disturbance, NPI - (Z=-2.15, p= .031), RBMPC (Z = -83.85, p< .001), agitation (Z= 6.75, p<.001) depression (Z=-15.99, p<.001) and anxiety (Z=-3.06, p= .002) decreased.	No long term follow up	Y	Y	Y	N	N
**Wells et al, 2000**	Four cognitive support units for PWD in nursing home section of US geriatric centre	5x 20-30 minute training in abilities focused morning care. Manualised, didactic and interactive. 20 - 30 minute reinforcement sessions fortnightly for 3 months, then monthly for 3 months.	16	20	TAU	28	20		At 6 month staff interacted with residents in more personal, relevant, sociable and flexible ways (t = -3.08, p = .005).		At 6 months resident's interactions were calmer and more positive (t = -2.07, p = .046), functioning improved (t = 2.37, p = .023) and agitation decreased (t = -2.12, p =0.041).	Y	Y	N	Y	N

***T-test not shown; Bold denotes that result is on an outcome identified as primary in study; CG= control group; IG=Intervention group; QOL-AD=Quality of life scale in Alzheimer's disease; QUALID=Quality of Life in Late-stage Dementia scale; PCC=Person Centred Care; RMBPC = Revised Memory and Behaviour Problems Checklist; TAU=Treatment as usual; WLC=Wait list control**

** Study validity was evaluated From Cooper et al 2014 Based on questions adapted from the Critical Appraisal Skills Programme checklist (http://www.sph.nhs.uk/sph-files/rct%20appraisal%20tool.pdf):

(1) Were participants randomised to intervention and control groups?

(2) Were patients and clinicians, as far as possible, 'masked' to treatment allocation? I have said yes if there was some attempt at blinding

(3) Were all patients who entered the trial accounted for and an intention to treat analysis used?

(4) Were all participants followed up and data collected in the same way?

(5) Was a power calculation carried out based on one of our outcomes of interest?

N given for participants with data at all time points.

Table 2: Methodological characteristics and quality ratings of lower quality qualitative studies

Study	Recruitment Source	Method	N	Type of intervention	Focus of analysis / key themes	Validity*					
						1	2	3	4	5	6
Brown-Wilson *et al.* (2013)	Staff in two English care homes participated in intervention. One provided follow up data.	Mixed methods practice development approach. Included questionnaires pre and post and case studies from workshops.	11 staff participated in workshops. 6 staff completed pre and 12 completed post questionnaire (2 completed both).	Facilitated workshops in relationship centred care (Senses framework).	How staff applied the Senses framework. Identified that staff felt enabled to create a sense of continuity and significance for the person with dementia.	Y	N	N	N	Y	Y
Chenoweth et al. (2015)	Family members, care managers, nurses and carers from Australian 38 long term care facilities.	Telephone survey with relatives, semi-structured interviews and analysis of facilitator field notes and resident care plans.	Survey with 73 relatives, interviews with 29 care managers and 70 nurses and care staff.	Person Centred Care intervention delivered using a 'train the trainers' model, training 'champions' to share approach via training, supervision, care planning and handover discussion. Person centred environment intervention delivered by two experts who trained unit managers to plan and implement environmental changes.	Improvements in care practices, improvements in resident agitation and well-being and factors which enabled or impeded implementation of the interventions.	Y	N	Y	N	Y	N
Cooney et al. (2014)	Staff, relatives and people with dementia from two public and two private Irish long term care homes.	In depth interviews.	11 residents, 5 relatives, 10 healthcare assistants, 9 nurses and 3 managers.	Reminiscence intervention – staff received structured reminiscence training and health care assistant / nurse dyads were assigned residents to complete life story and engage in four reminiscence sessions / week during 18-22 week intervention.	Core category of 'seeing me through my memories' was developed with three interrelated categories of 1. Seeing and knowing the person. 2. Reminiscence…a key. 3. Understanding and accommodating.	Y	Y	Y	N	N	N
Gotell *et al.* (2012)	Staff in one Swedish special care dementia unit.	Focus groups and semi-structured interviews post intervention.	17 staff participated in intervention. 9 took part in focus groups, 2 in joint interview and 1 in individual interview.	4 week intervention of staff singing during transfer situation. Included identifying of individualised songs and training delivered by singing instructors.	Staff experiences of singing to during transfer situations. Overall theme: Reciprocally spirited movements and disposition. Four subthemes: 1. Improved mutual transfer ability. 2. Enhanced mutual verbal and nonverbal communication; 3. Caregivers' new experiences, emotions, and moods. 4. Singing can be both straightforward and challenging.	Y	N	N	N	N	N
Guzman-Garcia *et al.* (2012)	Staff and people with dementia in two private English care homes.	Semi-structured interviews post intervention.	7 residents and 9 staff were interviewed.	Latin dance intervention 2x weekly 35 min sessions over 6 weeks. Led by therapist and facilitated by staff in homes.	Perceptions of the intervention and two explanatory models were developed, one for staff and one for people with dementia.	Y	N	Y	Y	N	Y
Hammar *et al.* (2010a) & Hammar *et al.*	Staff in two Swedish dementia nursing homes.	Focus groups with staff pre and post intervention.	Six care staff (3 in each home) 4 assistant nurses and 2 nurse aides.	Music intervention – Staff singing to or with people with dementia during morning care.	Hammar *et al.* (2010a) Staff perceptions of impact of intervention on residents. Pre intervention theme: Being in a different reality. Post intervention theme: Being present.	Y	N	Y	N	Y	Y

(2010b)					Hammar *et al.* (2010b) Staff perceptions of impact of intervention on themselves. The analysis resulted in two main themes: The first - Struggling for care in communion. The second - Consolidating care in communion.						
Hansebo & Kihlgren (2000)	Staff in three Swedish nursing home wards in different homes.	Pre and post intervention narratives collected from all staff on units about the life stories of residents.	30 participants (10 staff from each ward) provided narratives pre and post intervention.	Wards split into small caring teams and each team had 1 day training in use of a care planning assessment tool and were given 2hrs supervision by author / month over 1 year. Supervision taken over by nurse in caring team.	Examined differences in narratives pre and post and differences in narratives between Registered Nurses and Nursing Aides. Two main themes identified were: 1. The Perspective of the patient as a unique individual with resources and abilities despite limitations resulting from old age and dementia. 2. The perspective of the carer's approach to their patients and their duties.	Y	N	Y	N	Y	N
Hansebo & Kihlgren (2001)	Staff in one Swedish nursing home ward included in Hansebo *et al.* (2000).	Stimulated recall interviews of staff after watching videos of morning care interactions before, during and after the intervention.	N=4, 2 enrolled nurses and 2 nursing aides.	As in Hansebo *et al.* (2000).	Four main themes were: 1. Carers reflections, focusing on themselves. 2. Carers own caring philosophy. 3. Reflections focusing on the patient. 4. Reflections focussing on the context and the work itself in day to day shared life.	Y	N	Y	N	Y	N
Hansebo & Kihlgren (2004)	Staff in three Swedish nursing home wards.	Mixed methods evaluation. Included: Nursing records, patient life stories, videos, stimulated recall interviews and questionnaires.	50 Staff from 3 units contributed to different aspects of the evaluation.	As above	Triangulated data from range of sources to give overall impression of intervention.	Y	N	Y	N	Y	N
Kemeny *et al.* (2004)	Nursing staff / administrators and Nursing Aides in US long term care facility.	Post intervention focus groups.	Not provided. Held separate focus groups for nursing mentors, nursing aides and administrative staff.	Training and mentoring intervention for staff in managing challenging behaviour and using person centred care.	Staff understandings of changes in their behaviour post intervention. Explored differences in nurse mentors and nursing aides – Nursing aides were more likely to report sustained use of person centred approach and nursing mentors were less likely to be using mentoring skills.	Y	N	N	N	N	N
Lykkeslet et al. (2014)	Staff from one ward in a rural Norwegian nursing home.	Action research methodology including observations, focus groups and field note analysis.	Four staff members participated throughout two a year period and three temporary staff participated partially.	Sensory stimulation intervention which include didactic training, skills training, direct feedback on practice and a reflective practice group.	Care workers interactions with residents before, during and after the intervention period. Two main themes were 1. Gradually viewing symptoms as meaningful expressions. 2. Gradually realising the importance of human relationships.	Y	N	Y	N	Y	N
Moyle *et al.* (2013)	Staff, residents and family from three Australian long term care	Post intervention focus groups and semi structured interviews.	12 staff members in individual interviews and focus groups, 6 residents and 7 family	'Capabilities' model of dementia care: Included six two-hour education sessions over 2 months with staff, on-site	Implementation of the model and the impact of the intervention. Five main themes included: 1. General reflections on nursing care. 2. Implementation of the CMDC intervention. 3. Positive outcomes of the	Y	Y	N	N	N	N

	facilities.		members.	mentorship to consolidate skills and support implementation.	CMDC intervention. 4. Challenges in the implementation of the CMDC. 5. Difficulty sustaining care and tensions between participants' perspectives of care.						
Cooke *et al.* (2014)	As above	Mixed methods analysis of questionnaires and reflective diaries of facilitators.	Forty eight staff received the training with 2 facilitators /on site mentors	As above	Four themes emerged from analysis of reflective notes: 1. On-hand application and guidance. 2. Teaching and mentoring methods. 3. Visible progress. 4. Organisational support.	Y	N	Y	N	N	N
Rosvick *et al.* (2011)	Staff in two Norwegian dementia nursing homes.	Post pilot intervention focus groups.	11 registered nurses and 12 auxiliary nurses participated in four focus groups.	Pilot implementation of the 'VIPS' practice model. Model included: Regular structured team work, supervision and training and supportive management. Staff had manual and support materials and allocated different roles within the intervention.	Five main themes: 1. Legitimacy of the model was secured when central roles were held by nurses representing the majority of the staff. 2. The model facilitated the staff's use of their knowledge of PCC. 3. Support to the persons holding the internal facilitating roles in the model was needed. 4. The authority of the leading registered nurse in the ward was crucial to support the legitimacy of the model. 5. Form of organisation seemed to be of importance in how the model was experienced.	Y	N	Y	N	Y	N
Soderlund et al. (2012)	Staff in two Swedish nursing homes in. One nursing home took part in validation intervention the other was already using method.	Semi-structured interviews.	In intervention home 9 nurses were interviewed pre and post, 2 pre only and 3 post only. 9 nurses were interviewed in the other home.	Validation method intervention over one year; included ten days of training with between session supervision, practical training, documentation and written test.	Compared pre and post interviews and interviews across homes. Four main themes: 1. Being attentively present in the relationship. 2. Putting oneself into the resident's world. 3. Creating a trusting atmosphere by trusting the residents and trusting one's own abilities. 4. Difficulties in using the validation method.	Y	Y	N	N	Y	N
Soderlund et al. (2014)	Staff from three wards in nursing home that received validation method intervention.	As above	12 nurses interviewed post intervention.	As above	Nurses' experiences of using the validation method. Four key themes were 1. Being under extra strain. 2. Sharing experiences. 3. Improving in confidence in care situations. 4. Feeling uncertain about continuing.	Y	Y	N	N	Y	N
Teri *et al.* (2009)	Staff in eight assisted living facilities in three US states.	Train trainers evaluation included analysis of trainers' field notes, semi structured and questionnaires post intervention.	Three 'trainers' were trained and delivered the training in 8 sites. 40 unlicensed assistive staff and 36 leadership staff participated.	Trainers received 2 day training with follow up support. STAR delivered to staff with 1 four hour training session and 4 one hour individual sessions.<br>Three on-site support sessions were offered to leadership.	Five main themes identified: 1. Reactions to time pressures of the job. 2. Hesitation to try new strategies. 3. Conflicts with prior training and experiences. 4. Preconceived and unhelpful notions about the ''cause'' of resident behaviour. 5. Lack of awareness of the impact of their own behaviours.	Y	N	N	N	N	N

Vaiu-Guay *et al.* (2013)	Staff in 43 Canadian public and private long term care facilities.	Qualitative evaluation of post intervention survey data.	392 (94%) staff that participated in training completed the questionnaire.	Relationship based care training intervention included 2 days basic training, 0.5 day coaching and 3 hr consolidation meeting.	Analysis presents what aspects of the intervention were most and least useful and were hardest / easiest to integrate into care and why.	Y	N	Y	N/A	Y	Y
van Haeften-van Dijk et al. (2015)	Staff in from 21 Dutch Nursing Homes	Focus groups and semi-structured interviews.	12 stakeholders participated in semi-structured interviews and 35 staff participated in 5 focus groups.	The 'Veder Method' – A living room theatre based activity to improve person centred communication. Staff 1. Observed professional actors delivering the intervention2. Participate in a one-day training course 3. Deliver the intervention under observation and receive feedback.	Barriers and facilitators to the implementation process across existing conditions, preparation, execution and continuation phase. Organised into pre-defined theoretical framework.	Y	Y	N	Y	Y	N
Van Weert *et al.* (2004)	Staff in 6 Dutch nursing homes. (6/12 intervention wards in RCT).	Post intervention semi structured interviews and follow up meeting notes analysed.	80 caregivers attended training. Interviews were with six head nurses.	Implementation of snoezelen intervention included: 4 training sessions, study group in each home, individualised care planning, in house follow up meetings over 18 months with trainers, two meetings with managers to facilitate implementation.	Analysis presents: 1. Evaluation of the training programme. 2. Process evaluation of facilitating and hindering factors. 3. Evaluation of changes in daily care at caregiver, resident and organisational level.	Y	N	Y	N	N	N

*Study validity based on questions adapted from the Critical Appraisal Skills Programme checklist (http://www.sph.nhs.uk/sph-files/rct%20appraisal%20tool.pdf): Adapted from Mukadam, N. et al., Int J Geriatr Psychiatry, 2011; 26: 12–20, Lord, K. et al., Int Psychogeriatrics, 2015, 27: 1301-1312.

Quality assessment tool for qualitative studies
(1) Were the aims of the research clearly stated?
(2) Was a clearly defined method of recruitment used and explicit inclusion/exclusion criteria described?
(3) Was the process of data collection explained clearly? Was data collection standardised?
(4) Did the researchers attain saturation of data?
(5) Was the process of data analysis sufficiently rigorous, i.e. ≥2 raters, some method of resolving discrepancies?
(6) Have the findings been validated by participants?
